# DAT1 and Its Psychological Correlates in Children with Avoidant/Restrictive Food Intake Disorder: A Cross-Sectional Pilot Study

**DOI:** 10.3390/bs11010009

**Published:** 2021-01-14

**Authors:** Silvia Cimino, Eleonora Marzilli, Alessandra Babore, Carmen Trumello, Luca Cerniglia

**Affiliations:** 1 Department of Dynamic and Clinical Psychology, Sapienza, University of Rome, 00185 Roma, Italy; silvia.cimino@uniroma1.it (S.C.); eleonora.marzilli@uniroma1.it (E.M.); 2 Department of Psychological, Health and Territorial Sciences, University “G. d’Annunzio”, 66100 Chieti, Italy; a.babore@unich.it (A.B.); c.trumello@unich.it (C.T.); 3 Faculty of Psychology, International Telematic University Uninettuno, 00186 Roma, Italy

**Keywords:** avoidant/restrictive food intake disorder, diagnostic subtypes, dopamine transporter, genotype, methylation, gene-environment interaction, psychopathological symptoms

## Abstract

International research has underlined the role played by children’s and maternal psychopathological symptoms on the onset of avoidant/restrictive food intake disorder (ARFID) in early childhood. No study has considered the possible interplay between children’s dopamine transporter (DAT1) genotype and methylation, dysregulation problems and maternal psychopathological risk. This study aimed to investigate the complex relationship between these variables, considering the possible mediation role played by children’s DAT1 methylation on the relationship between mothers’ psychopathological risk and children’s dysregulation problems, moderated by children’s DAT1 genotype. Our sample consisted of 94 early children and their mothers, divided into four subgroups, based on children’s ARFID subtypes (irritable/impulsive (I/I), sensory food aversions (SFA), post-traumatic feeding disorders subtypes (PTFD), and a non-clinical group (NC)). We addressed children’s dysregulation problems and maternal psychopathological risk, and collected children’s DNA through buccal swabs. Results showed that children’s 9/x genotype was associated with PTFD and NC groups, whereas the 10/10 genotype was associated with the SFA group, with large effect size. There were significant large differences in the study groups on children’s DAT1 total methylation, children’s dysregulation problems, and maternal psychopathological risk. Children’s DAT1 methylation did not mediate the relationship between mother’s psychopathological risk and children’s dysregulation problems, but there was a significant large direct effect. Children’s 9/x genotype moderated the relationship between maternal psychopathological risk and children’s DAT1 methylation but, respectively, with a large and small effect. Our pilot study suggested that the relationship between children’s DAT1 genotype and methylation, dysregulation problems, and maternal psychopathological risk has a crucial contribution to ARFID.

## 1. Introduction

### 1.1. Dopamine Transporter Gene and Feeding Disorders

Central dopaminergic mechanisms play a major role in reward-motivated behavior involved in eating and food choices [1,2], and are associated with positive hedonic processes related to food [3,4,5]. Dopamine (DA) neurotransmission is regulated primarily by DA transporter (DAT), which removes DA from the synaptic cleft back into the presynaptic neurons. The gene that encodes the DAT protein, known as DAT1, has a variable number of tandem repeats (VNTR) of 40 bp (3–11 repeats) in the 3′ untranslated region (UTR). A greater gene expression has been shown associated with DAT1 10/10 genotype [6,7]. Thus, DAT1 is implicated in a number of DA-related disorders (including attention-deficit/hyperactivity disorder (ADHD), bipolar disorder, depression, and substance dependence) [8,9,10]. Dysregulation of DA signaling (ascribable to either DAT1 polymorphisms or hyper/hypomethylation of DAT1 promoter) is also likely to be involved in eating disorders (EDs), including bulimia nervosa and binge eating disorder [11,12]. The higher frequency of the short allele (i.e., 7 or 9 repeats, in comparison to the long allele, 10 or 11 repeats) in the DAT1 gene reported by Shinohara and colleagues [13] suggests decreased DAT function in patients who have EDs with binge-eating behaviors. Moreover, increased DAT mRNA levels have been reported by Frieling and colleagues [14] on bulimic patients. Despite the above literature, no studies to our best knowledge focused on samples of young children. Interestingly, however, one study [15] found an association between ADHD symptoms and disordered eating in young children, suggesting that the DA path could be involved in the underpinning mechanism explaining both difficulties. Previous research has shown associations between ADHD in children and DAT1 polymorphism and methylation [16,17,18,19]. Moreover, undernutrition has been shown to be associated with epigenetic changes in humans and DNA polymorphisms [20,21].

### 1.2. Bio-Psycho-Social Correlates of Avoidant/Restrictive Food Intake Disorder in Early Childhood

Recent studies, rooted in the developmental psychopathology framework [22], have employed complex bio-psycho-social approaches evaluating the interaction of environmental (maternal psychopathology) and individual factors (bio/genetic characteristics of offspring), especially in samples of early children [23,24], also reporting significant associations between children’s DAT1 methylation and polymorphism with maternal psychopathology [18,19,25]. Notwithstanding these important results, no previous study evaluated DAT1 polymorphism and methylation status in children with avoidant/restrictive food intake disorder (ARFID), a new diagnostic category included in the section of Feeding and Eating Disorders of the Fifth Edition of the Diagnostic and Statistical Manual of Mental Disorders (DSM-5) [26]. Children with ARFID fail to eat adequate intake of food and required nutrients manifesting impaired social and psychological functioning [26,27]. Very importantly, ARFID encompasses different clinical manifestations, and some studies have used the term subtypes [28,29,30,31], according to the nature of their eating restrictions. One possibility is that children may be highly selective and avoid specific foods due to their smell, texture, or appearance, a subtype named sensory food aversion (SFA) [27,32]. Other patients, named with the clinical labels of post-traumatic feeding disorder (PTFD) subtype [30,32], may show food avoidance after distressing experiences involving oropharyngeal and gastrointestinal tract. Moreover, some children with ARFID may show characteristics of impulsiveness, irritability, and difficult to console and to engage during meals [26,27,33]. It has also been reported significant association between impulsive/hyperactive difficulties and food refusal behaviors among preschooler children [15,34], as well a history of selective eating during the early childhood of children with ADHD [35]. Although difficulties in emotional/behavioral regulation have been posited as correlated with EDs in adolescents and adults, very few studies have focused on children with ARFID [36,37,38], considering its subtypes [31,39]. Maternal psychopathology is also frequently associated with internalizing and externalizing, emotional/behavioral dysregulation, and eating/feeding disorders in children [28,39,40]. Moreover, although it has been suggested that the influence of the quality of the emotional environment provided by parents on children’s psychological well-being could be mediated by DNA methylation mechanisms [41,42] that, in turn, could be moderated by the child’s genotype [43,44], no study to date has assessed the role played by DAT1 in these processes.

### 1.3. The Present Study

Based on the above premises, this pilot study aimed at increasing knowledge of these processes among children with three specific ARFID sub-groups (impulsive/irritable (I/I), SFA, PTFD), compared with a control group. In accordance with previous literature [28,29,30,31], we have chosen to focus on three specific clinical manifestations of ARFID, defining them in terms of subtypes. In particular, we wanted to explore: (a) the possible association between children’s DAT1 genotype and ARFID diagnosis; (b) the possible significant differences on children’s DAT1 total methylation, their dysregulation problems, and maternal psychopathological risk; and (c) the possible mediation role played by children’s DAT1 methylation on the relationship between mothers’ psychopathological risk on children’s dysregulation problems, moderated by children’s DAT1 genotype.

## 2. Materials and Methods

### 2.1. Study Design

A cross-sectional study was carried out involving N = 191 children and their mothers, recruited through public and private kindergartens, mental health clinics, and pediatric hospitals in central Italy. Mothers were contacted by expert psychologists who explained the phases and the scope of the study. All mothers who decided to participate in the study signed an informed consent form, in which the aims of the study were explained in detail. The study was approved by the Ethical Committee of the Psychology Faculty at the International Telematic University Uninettuno (n. 2018/3), and it is in line with the Declaration of Helsinki (see Supplementary Materials).

### 2.2. Procedure

Children’s biological samples were collected through buccal swabs (Isohelix Swab Pack) from which it is possible to extract the DNA present in the epithelial cells and subsequently examine genetic polymorphism and methylation status of DAT1. Mothers were previously informed that, for at least 1 h before collecting children’s salivary sample, they should not have eaten (including chewing gum, sweets, etc.), drunk (with the exception of water), or brushed their teeth. Once the buccal swabs were collected, they were slightly chilled by normal ice (+4 °C). After the administration of tampons, mothers filled out self-report and report-form questionnaires (described below), for the assessment of children’s emotional-behavioral functioning and their own psychopathological symptoms. The administration of buccal swabs and questionnaires was made by expert psychologists inside a room made available, respectively, by kindergartens and mental health clinics.

### 2.3. Participants

To recruit clinical cases we used consecutive sampling, whereas children of the control group were randomly selected from each kindergartens. Inclusion criteria for the clinical groups were the presence of a diagnosis of one of the three ARFID subtypes (I/I, SFA, PTFD), without a comorbid disorder. The diagnosis subtypes were made by two clinicians, independently (Cohen’s k = 0.81), on the basis of the clinical presentations outlined in the DSM-5 [26,33] and in DC 0-3R [27]. The inclusion criteria of the control group were: age range of children from 24 to 36 months; absence of physical or mental disorders in children and/or their mothers; mothers were biological parents of the child. 

### 2.4. Measures

For the assessment of children’s emotional-behavioral self-dysregulation, mothers were administered the Child Behavior Check-List/1½–5 (CBCL 1½–5) [45,46]. The CBCL 1½–5 is a 99-item informant-report questionnaire through which the parent is asked to answer on a three-point Likert scale (from 0 = “not true” to 2 = “very true or often true”). The scores of items were grouped on seven syndrome scales: Emotionally Reactive, Anxious/Depressed, Somatic Complaints, Withdrawn, Attention Problems, Aggressive Behavior, and Sleep Problems. The CBCL Dysregulation Profile (DP) is composed of the sum of the raw scores of the following syndrome scales: Anxious/Depressed, Attention Problems, and Aggressive Behavior [47]. The scale showed very good internal consistency, with a Cronbach’s α value of 0.79 in this study. For the assessment of maternal psychopathological risk, mothers filled out the Symptom Check-List-90 item-Revised (SCL-90-R) [48]. The SCL-90-R is a 90-item self-report questionnaire aimed at evaluating psychological symptoms and psychological distress in adults. Items are measured on a Likert scale ranging from 0 (not at all) to 4 (extremely). It is composed of nine primary dimensions (Somatization, Obsessive-Compulsivity, Interpersonal Sensitivity, Depression, Anxiety, Hostility, Phobic Anxiety, Paranoid Ideation, and Psychoticism), and to provide the severity and degree of psychological distress, it is possible to calculate a Global Severity Index (GSI), used for the aim of this study. The Italian validation [49] showed good reliability in terms of internal consistency (Cronbach’s α = 0.70–0.96), that in this study was also adequate (Cronbach α = 0.83).

### 2.5. DNA Isolation and Genotyping

DNA extraction from the buccal wall cells was performed using the Buccal-Prep Plus DNA isolation (Isohelix), following the manufacturer’s instructions. Information of the DAT1 gene was determined from the DAT1 gene sequence (NG_015885.1). Specifically, to identify the DAT1 polymorphism we amplified the repeated sequence of the 3 ‘untranslated (3′-UTR) region, by the polymerase chain reaction (PCR) technique [50]. Based on previous studies in the field of developmental psychopathology, which compared children with at least one allele 9-repeated (9/*x*; 9/9, 9/10) [17,18,19,51,52], results are reported considering the absence or presence of the 9/*x* allele (10/10 vs. 9/9, 9/10). Allelic distributions were consistent with Hardy–Weinberg equilibrium for each group (I/I, χ^2^ = 2.13, *p* = 0.14; SFA, χ^2^ = 0.57, *p* = 0.45; PTFD, χ^2^ = 2.68, *p* = 0.10; NC, χ^2^ = 2.83, *p* = 0.09).

### 2.6. Analysis of DNA Methylation

The DNA extracted from the buccal swabs was further processed to evaluate the amount of methylation in the 5′-UTR sequence of DAT1. The amount of methylation was determined in six specific CpG sites (named M1, M2, M3, M5, M6 and M7), as suggested by previous studies in the field of developmental psychopathology [17,18,19]. The following primers (5′-3′) were used to amplify the gene: Fwd, AGCTACCATGCCCATCCCTA TGTGGG; Rev, ATCAGCACTCCAACCCAACCCAAC. The DNA was amplified with the PyroMark PCR kit (Qiagen, Hilden, Germany) according to the manufacturer’s protocol. The PCR conditions were as follows: 95 °C for 15 min, followed by 45 cycles of 94 °C for 30 s, 56 °C for 30 s, 72 °C for 30 s, and 72 °C for 10 min. PCR products were verified by agarose electrophoresis. The methylation level was analyzed using the PyroMark Q24 software (Qiagen, Hilden, Germany), which calculates the methylation percentage using the formula mC/(mC + C) for each CpG site, allowing quantitative comparisons (mC is the methylated cytosine and C is unmethylated cytosine). Finally, we calculated the Total DNA methylation (overall 6 CpG sites) of this promoter region, used for statistical analyses. Details on the sequence and the pyrosequencing assay (PM00022064) are available on the Qiagen web site (www.qiagen.com).

### 2.7. Statistical Analyses

Preliminary analyses were performed using descriptive statistics (frequencies, percentages, and mean scores). The association between children’s DAT1 genotype and children’s diagnoses was examined using chi-square analysis, with the effect size represented using Cramer’s V. Information about the frequency distribution of the variables was reported in contingency table. If the value of an adjusted residual was more than 1.96, the number of cases in that cell was significantly larger than expected. Differences between the four subgroups on children’s levels of DAT1 total methylation, their emotional-behavioral self-dysregulation, and mothers’ psychopathological risk were examined using univariate analyses of variance (ANOVAs), and partial eta squared (ηp^2^) were reported as a measure of effect size. Finally, on the total sample, we explored possible mediation effects of children’s DAT1 total methylation on the relationship between maternal GSI and children’s score of CBCL DP, moderated by children’s DAT1 genotype. We reported R^2^ as a measure of effect size. Moderated mediation analyses were performed using Hayes’s [53] PROCESS macro (Model 59). Indirect (i.e., mediating) effects were evaluated with 95% bias-corrected confidence intervals based on 10,000 bootstrap samples. Confidence intervals (CI) that do not include zero indicate effects that are significant at α = 05. All analyses were performed using SPSS software, Version 25.0.

## 3. Results

### 3.1. Sample Characteristics

From the total sample, N = 39 families refused to participate in the study. Moreover, were excluded families in which children had comorbidity between SFA and infantile anorexia (N = 9) and/or had comorbidity between PTFD and SFA (N = 2), and/or in which a member (the child and/or the mother) was under pharmacological or psychological treatment (N = 14), or suffering from cognitive, neurological, and/or physical problems (N = 21). Moreover, N = 8 families were excluded because mothers did not complete all measures, and N = 4 because it was not possible to analyze DAT1 due to errors in gathering biological samples.

The final sample consisted of N = 94 children aged from 24 to 36 months (M = 29 months, SD = 3.14; 51.1% males) and their mothers (M = 32.3; SD = 4.2), divided into four subgroups, based on children’s diagnosis: (i) I/I group, composed by children with ARFID I/I subtype diagnosis, and their mothers (N = 23); (ii) SFA group, composed by children with ARFID SFA subtype diagnosis, and their mothers (N = 23); (iii) PTFD group, composed by children with ARFID PTFD subtype diagnosis, and their mothers (N = 23); and (iv) NC group, composed by children with no diagnosis, and their mothers (N = 25). Most mothers had high school (78.5%) or university (20.1%) education, and only 1.4% of mothers had only middle school education. The vast majority of mothers had average socioeconomic status (94% had an average income of 25,000–30,000 Euros per year). All mothers were the biological parent of the children and were Caucasian.

### 3.2. Association between Children’s Dopamine Trasnporter Genotype and Children’s Diagnoses

Chi-square analysis showed a significant association between children’s DAT1 genotype and children’s diagnoses, χ^2^(3, N = 94) = 40.05, *p* < 0.0001, with a large effect size (Cramer’s V = 0.70). ARFID I/I subtype was not associated with any specific DAT1 genotype. SFA subtype was associated with 10/10 genotype, whereas PTFD and NC groups were associated with 9/x genotype (Table 1).

### 3.3. Children’s Dopamine Transporter Methylation, Emotional-Behavioral Dysregulation, and Maternal Psychopathological Risk in the Four Groups

Possible differences in the children’s DAT1 total methylation levels, children’s dysregulation (CBCL DP), and maternal psychopathological risk (SCL-90/R GSI) were examined using ANOVAs, considering the group (I/I, SFA, PTFD, NC) as independent variables. Levene’s test showed the presence of a non-homogeneity of variance (Levene test, *p* < 0.05) for all dimensions considered. Thus, Welch’s test was conducted. The results showed that the four groups were significantly different on children’s levels of DAT1 total methylation (F(3,46) = 52.76, *p* < 0.0001, ηp^2^ = 0.71), the score of CBCL DP (F(3,46) = 266.06, *p* < 0.0001, ηp^2^ = 0.85), and mothers score on GSI (F(3,44) = 791.57, *p* < 0.0001, ηp^2^ = 0.95), with large effect sizes. In particular, Dunnett T3 post-hoc test showed that children of the I/I group had higher levels of DAT1 total methylation than other groups (*p* < 0.0001). Moreover, children with an SFA diagnosis and with no diagnosis had higher levels of DAT1 methylation than children with PTFD diagnosis (*p* < 0.0001). Finally, children with PTFD had lower levels of DAT1 total methylation than other groups (*p* < 0.0001). As regards children’s emotional and behavioral self-dysregulation, children of the NC group had lower scores than other groups in CBCL DP (*p* < 0.0001). Moreover, children with I/I and with a PTFD diagnosis had higher levels of DP than the SFA and NC groups (*p* < 0.0001). There were no significant differences between children of the I/I and PTFD groups (*p* > 0.05). Finally, mothers of children with no diagnosis reported lower scores on GSI than other groups (*p* < 0.0001), whereas mothers of children with a I/I diagnosis reported higher scores compared to mothers of other groups (*p* < 0.0001). Mothers of the PTFD group had higher scores of GSI than mothers of the SFA group (*p* < 0.0001) (Table 2).

### 3.4. Children’s Dopamine Transporter Methylation as a Mediator of the Association between Maternal Psychopathological Risk and Children’s Dysregulation, Moderated by Children’s Genotype

**Figure 1:**
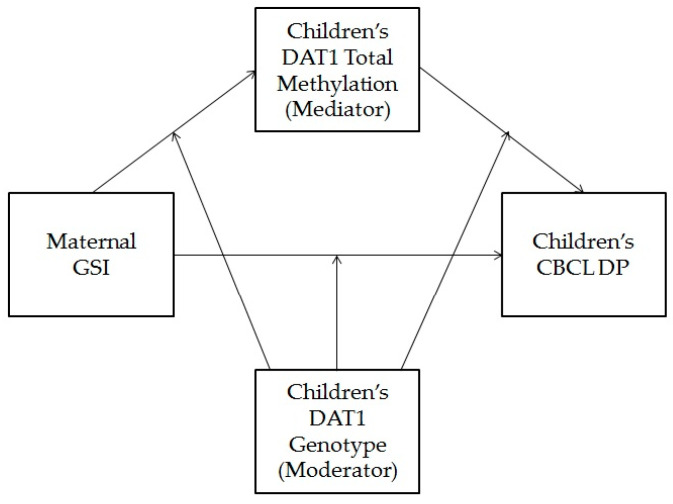
Diagram of moderated mediation model with children’s DAT1 total methylation as the mediator of the relationship between maternal global severity index (GSI) and Children CBCL/1½-5 Dysregulation Profile (CBCL DP), with children’s DAT1 genotype as the moderator.

Finally, in order to verify whether children’s DAT1 total methylation mediates the effect of mothers’ GSI on children’s DP by children’s DAT1 genotype we conducted moderated mediation analysis. For this aim, we considered the total sample. Figure 1 shows the proposed moderated mediation model.

Results showed that the significant direct effect of GSI on children’s DAT1 total methylation was significant. Moreover, there were significant direct effects of mothers’ psychopathological risk and children’s DAT1 genotype on children’s CBCL DP. The interaction effect of children’s DAT1 genotype with children’s DAT1 total methylation was also significant. To probe this significant interaction, conditional effects were evaluated using the PROCESS macro [53]. Results showed that the level of children’s DAT1 total methylation was a significant negative predictor of the score of CBCL DP, but only for children with 9/x genotype (B = −2.57, SE = 0.64, *p* < 0.0001; 10/10, B = 3.90, SE = 2.78, *p* = 0.16), but with a low effect size (F(1,88) = 5.14, *p* = 0.02, R^2^-chng = 0.01). Moreover, results of conditional direct effect of maternal GSI on CBCL DP showed that mother’s psychopathological risk significantly predicted children’s CBCL DP for both children’s genotypes (*p* < 0.0001), with a large effect size. Finally, the indirect effect of mothers’ GSI on children’s DP through levels of children’s DAT1 total methylation was not significant (the CI included zero) (Table 3).

## 4. Discussion

This pilot study aimed to investigate children’s levels of DAT1 methylation, their emotional and behavioral dysregulation, and psychopathological risk of their mothers, in a sample of young children with three different ARFID subtypes (i.e., I/I, SFA, PTFD). Our preliminary analyses, focused on the specific characteristics of the three ARFID subtypes, have underlined peculiar features associated with each diagnosis.

### 4.1. Impulsive/Irritable Subtype

Regarding the I/I subtype, results showed no significant association with children’s DAT1 genotype, although the majority of this group (82.6%) had 9/x genotype. Moreover, children with I/I had the highest levels of DAT1 methylation, which are generally associated with reduced levels of transcription and gene expression, resulting in lower availability of DAT and greater levels of extracellular DA [54,55]. Consequently, our results suggested that children with I/I may have dopaminergic hyper-functioning that, in turn, have been associated with children’s impulsive behaviors [56], as well as with adolescent’s and adults’ restraint eating and bulimic behaviors [57,58]. They also showed high levels of emotional-behavioral dysregulation, and their mothers showed the greatest psychopathological risk. Previous studies have shown that early food avoidant behaviors may be associated with impulsivity behaviors in preschooler children [15] and hyperactive symptoms over time [34,35], and recent studies by Peyre and colleagues [59] and by Katsuki and colleagues [60] have evidenced a key role played by CBCL DP in ADHD. The present study also suggested that the I/I subtype may be a higher risk for dysregulation problems, in line with recent literature [31].

### 4.2. Sensory Food Aversions Subtype

With regard to SFA diagnosis, we found a significant association with 10/10 genotype, with large effect size, and DAT1 methylation was at a medium level (higher than PTFD, lower than I/I, and similar to NC). In addition, these children reported the lowest dysregulation problems compared to other clinical groups and, in turn, their mothers’ showed the lowest psychopathological risk than mothers of other ARFID groups. These results are in line with the study by Lucarelli and colleagues [28] and Cerniglia and colleagues [31] that have evidenced lower psychopathological problems in children with SFA and among their mothers, compared to other ARFID subtypes.

### 4.3. Post Traumatic Feeding Disorder Subtype

Finally, PTFD diagnosis was associated with 9/x genotype, as well as the NC group, with a large effect size. In this context, it is important to note that although previous studies have reported significant association between DAT1 and a wide range of children’s psychopathologies [8,17,61,62,63], only few studies have focused on feeding disorders [13,64]. Our study is one of the first, supporting the recent evidence that individual genetic features could make the child more susceptible to both adverse and supportive influences [62,65,66], as a possible explanation of the associations found between genotype 9/x with both PTFD and NC group. Children with PTFD diagnosis also reported the lowest levels of DAT1 methylation, in line with previous studies on traumatized individuals that found low levels of DA in the brain among this population [67]. They also had the highest scores of CBCL DP, and their mothers had the highest psychopathological risk. The research on the impact of early traumatic experiences has evidenced a significant association with emotional and behavioral dysregulation difficulties [68,69]. This study suggests that even a child exposed to an early food-related trauma may be at higher risk to develop dysregulation problems. Moreover, our findings are in accordance with previous studies that have shown the highest psychopathological risk in children with PTFD and among their mothers compared to other ARFID diagnosis [28,31].

### 4.4. The Complex Interplay between the Variables

Our last aim was to verify the possible mediation role played by children’s DAT1 methylation on the relationship between maternal psychopathological risk and children’s dysregulation, and if these relationships were moderated by children’s DAT1 genotype. To this end, we considered the total sample. However, our results showed no significant indirect effect of mothers’ GSI on children’s DP through levels of children’s DAT1 total methylation. We found a significant association between maternal GSI and children’ DAT1 methylation, as well as a direct and moderated association with children’s DAT1 genotype, in accordance with previous studies [18,19]. Moreover, both maternal psychopathological risk and children’s DAT1 genotype (i.e., 9/x genotype) were significantly and directly associated with children’s emotional-behavioral self-dysregulation, but the interactive effect was not significant. At the same time, 9/x genotype was shown to have a positive contribution on the scores of CBCL DP. This result is in line with our preliminary results on the different ARFID subtypes, which evidenced high levels of dysregulation problems among children with PTFD and I/I subtypes, which in turn were carriers of 9/x genotype. Regarding the relationship between children’s DAT1 methylation and children’s levels of DP, only the moderated association by children’s DAT1 genotype was significant, but with a low effect size. The level of children’s DAT1 methylation was significantly and negatively associated with the score of CBCL DP, but only for children with 9/x genotype. Given that the literature and our previous findings have suggested that psychopathological symptoms are associated with hypo-methylation of DNA (the PTFD group reported the lowest methylation levels, the highest scores on CBCL DP, and association with 9/x genotype), it would seem that the 9/x genotype confers an additional risk with respect to the effects of methylation on dysregulation problems. A recent study by Cimino and colleagues [19] also showed a moderating effect of children’s DAT1 genotype on the relationship between maternal psychological profiles and children’s methylation of DAT1, considering a sample of school-aged children of the general population.

### 4.5. Limitations and Strengths

This pilot study has some limitations. First of all, the small sample size of each subgroup compared to the standards of genetic studies, which implies a limited statistical power and does not allow to generalize our results. Furthermore, due to the cross-sectional nature of the study design, it is suggested to take with caution the preliminary causal links found. Consequently, further studies with larger study populations and within longitudinal study design are needed to support our preliminary findings. In addition, we assessed maternal psychopathological risk and children’s emotional-behavioral dysregulation, respectively, through self-report and report-form measures. Although the tools chosen are widely validated and used in the literature, information provided may be influenced by perception biases, and should be interpreted with caution. Further studies should evaluate these variables through more objective and robust methodologies, such as clinical interview and observational methodologies. Moreover, we didn’t consider the possible role played by confounder variables, such as: father’s psychopathological risk, which literature has evidenced to be implicated in children’s emotional-behavioral dysregulation, offspring’s feeding disorders [70,71,72], as well as children’s DAT1 methylation [18,19], which may act as an additional moderator on the relationship between maternal psychopathological risk and children’s outcomes; social support and marital adjustment, which are widely shown to be associated the psychological well-being of both parents and children [73,74]; the quality of feeding interactions, which may act as mediator on the relationship between mothers and children emotional-behavioral and feeding problems [71,75], with bidirectional effects [76]; finally, maternal DAT1 genotype, which in turn can influence maternal psychological profiles, as well as the quality of the relationship with their child, and the consequences on his/her adjustment [77,78]. Nonetheless, the present pilot study has several strengths. In fact, it was the first to explore the role played by DAT1 genotype, DAT1 methylation, and dysregulation problems among three different subtypes of ARFID, also considering the possible moderated mediation role played by levels of children’s DAT1 methylation on the relationship between maternal psychopathological risk and emotional-adaptive functioning of the child. Furthermore, the study added to the previous literature new support on the risk and/or protective factors, in children and their mothers, at the basis of ARFID in early children, which can be evaluated for the planning of more targeted and effective intervention strategies. Interestingly, the significant associations that we found between ARFID diagnosis, children’s DAT1 genotype and methylation, dysregulation problems, and maternal psychopathological risk had large effect sizes. The only one significant effect found in a low size was the moderated effect of children’s genotype on the association between children’s DAT1 methylations and their dysregulation problems. This finding may suggest that the influence of children’s DAT1 genotype is higher by itself and in response to the risk exerted by maternal psychopathological risk.

## 5. Conclusions

Overall, our preliminary findings have supported the importance to considering the complex interaction between DAT1 gene and environment provided by mothers, both in terms of genotype-environment interactions and the possible methylation mechanisms involved. The link that has emerged between maternal psychopathological risk, children’s DNA methylation, and their regulatory difficulties further suggests the importance of intervening also on the maternal difficulties of children with ARFID. In fact, recent evidence has shown that intervention strategies aimed at reducing risk factors and promoting protective factors can reverse the epigenetic signs, modifying that sequence of events called developmental cascade [79], in order to obtain the maximum effect on the change of a negative cascade in a positive one [80]. In contrast, epigenetic modifications resulting from exposure to environmental risk factors, and their effects on child’s emotional-behavioral functioning, can last well beyond the end of exposure itself [42,81]. Our study is in line with these evidences, supporting the importance of an assessment and intervention approach that takes into account not only the child’s feeding difficulties, but also their regulatory difficulties within the more complex family context [36,82].

## Figures and Tables

**Table 1:** Association between children’s dopamine transporter (DAT1) genotype and children’s diagnoses.

		Children’s Diagnoses				
DAT1 Genotype		I/I	SFA	PTFD	NC	Total
9/*x*	N (%)	19 (82.6)	4 (17.4)	21 (91.3)	24 (96)	68
	Exp. Val.	16.6	16.6	16.6	18.1	68.0
	St. R	1.3	−6.8	**2.3**	**3.1**	
10/10	N (%)	4 (17.4)	19 (82.6)	2 (8.7)	1 (4)	26
	Exp. Val.	6.4	6.4	6.4	6.9	26.0
	St. R	−1.3	**6.8**	−2.3	−3.1	
Total	N	23	23	23	25	94

9/*x* = 9/9, 9/10 genotypes; I/I = Avoidant/restrictive food intake disorder (ARFID) irritable/impulsive subtype group; SFA = ARFID sensory food aversions subtype group; PTFD= ARFID post-traumatic feeding disorder group; NC = Non-clinical group; Exp. Val = Expected values; St. R = Standardized adjusted residuals. All bold values are statistically significant.

**Table 2:** Means (M) and standard deviations (SD) in the four groups.

	Children’s Diagnosis							
	I/I		SFA		PTFD		NC	
Variable	M	SD	M	SD	M	SD	M	SD
DAT1 Total methylation	8.29 ^a^	1.07	6.01 ^b^	0.93	4.08 ^c^	1.18	6.08 ^b^	0.54
CBCL DP	33.69 ^a^	5.53	18.39 ^b^	5.67	35.43 ^a^	5.05	7.12 ^c^	2.83
SCL-90/R GSI	1.95 ^a^	0.22	0.60 ^b^	0.10	1.54 ^c^	0.17	0.18 ^d^	0.06

^a,b,c,d^ Means that do not share a letter are significantly different. I/I = ARFID irritable/impulsive subtype group, SFA = ARFID sensory food aversions subtype group, PTFD = ARFID post-traumatic feeding disorder group, NC = Non-clinical group.

**Table 3:** Results of moderated mediation analyses.

**Variable**		**B(SE)**	**LLCI**	**ULCI**
DAT methyl.				
	GSI	0.23 (0.11) *	**0.01**	**0.45**
	DAT1 genotype ^†^	−0.14 (0.26)	−0.66	0.38
	GSI x DAT1 genotype ^†^	−0.31 (0.32)	−0.96	0.33
		*R*^2^ = 0.04		
		F (3,90) = 1.51		
CBCL DP				
	GSI	11.26 (0.73) **	**9.80**	**12.71**
	DAT methylation	−1.06 (0.81)	−2.68	0.56
	DAT1 genotype ^†^	−3.39 (1.61) *	**−6.60**	**−0.19**
	GSI x DAT1 genotype ^†^	0.84 (2.35)	−3.83	5.52
	DAT methyl x DAT1 genotype ^†^	−6.48 (2.85) *	**−12.15**	**−0.80**
		*R*^2^ = 0.79		
		F (5,88) = 69.98 ***		
**Conditional Direct Effect at Specific Levels of Moderator**				
**Predictor**	**Moderator**	**Direct Effect (SE)**	**LLCI**	**ULCI**
GSI	10/10	10.61 (2.25) **	**6.12**	**15.10**
	9/9, 9/10	11.45 (0.66) **	**10.13**	**12.77**
		*R^2^* = 0.75F (3,90) = 93.75 **		
**Conditional Indirect Effect at Specific Levels of Moderator**				
**Mediator**	**Moderator**	**Indirect Effect (BootSE)**	**LLCI**	**ULCI**
DAT methyl.	10/10	1.85 (2.52)	−3.20	6.74
	9/9, 9/10	−0.41 (0.33)	−1.17	0.14

^†^ The contrast group is 10/10 genotype. * *p* < 0.05, ** *p* < 0.0001. SE = Standard error; LLCI = Lower level confidence interval; ULCI = Upper level confidence interval; BootSE = Boot-strapped standard error. All bold values are statistically significant.

## Data Availability

Data is available on request to the corresponding author.

## Supplementary Materials

Supplementary Materials are available online at https://www.mdpi.com/2076-328X/11/1/9/s1.
